# Evaluation and application of the CROPGRO-soybean model for determining optimum sowing windows of soybean in the Nigeria savannas

**DOI:** 10.1038/s41598-022-10505-4

**Published:** 2022-04-25

**Authors:** J. F. Bebeley, A. Y. Kamara, J. M. Jibrin, F. M. Akinseye, A. I. Tofa, A. M. Adam, N. Kamai, R. Solomon

**Affiliations:** 1 Department of Agronomy, Bayero University Kano, Kano, 700241 Nigeria; 2 International Institute of Tropical Agriculture (IITA), Kano, Nigeria; 3 Centre for Dryland Agriculture, Bayero University, Kano, Nigeria; 4 Department of Soil Science, Bayero University Kano, Kano, 700001 Nigeria; 5 International Crop Research Institute for the Semi-Arid Tropic (ICRISAT), Kano, Nigeria

**Keywords:** Plant sciences, Environmental sciences

## Abstract

Soybean production is limited by poor soil fertility and unstable rainfall due to climate variability in the Nigeria savannas. There is a decline in the amount and duration of rainfall as one moves from the south to north of the savanna zones. The use of adapted soybean varieties and optimum sowing windows are avenues to increase productivity in the face of climate variability. Crop simulation models can be used as tools for the evaluation of alternative management options for a particular location, including fertilizer application rates, plant density, sowing dates and land use. In this study, we evaluated the performance of the Cropping System Model (CSM)-CROPGRO-Soybean to determine optimum sowing windows for three contrasting soybean varieties (TGX1835-10E, TGX1904-6F and TGX1951-3F) cultivated in the Nigeria savannas. The model was calibrated using data from ten field experiments conducted under optimal conditions at two sites (BUK and Dambatta) in Kano in the Sudan savanna (SS) agro-ecology over four growing seasons. Data for model evaluation were obtained from independent experiment for phosphorus (P) response trials conducted under rainfed conditions in two locations (Zaria and Doguwa) in the northern Guinea savanna (NGS) zone. The model calibration and evaluation results indicated good agreement between the simulated and observed values for the measured parameters. This suggests that the CROPGRO-Soybean model was able to accurately predict the performance of soybean in the Nigeria savannas. Results from long-term seasonal analysis showed significant differences among the agro-ecologies, sowing windows and the soybean varieties for grain yield. Higher yields are simulated among the soybean varieties in Zaria in the NGS than in Kano the SS and Jagiri in the southern Guinea savanna (SGS) agro-ecological zones. Sowing from June 1 to July 5 produced optimal yield of TGX1951-3F and TGX1835-10E beyond which yield declined in Kano. In Zaria and Jagiri the simulated results show that, sowing from June 1 to July 12 are appropriate for all the varieties. The variety TGX1951-3F performed better than TGX1904-6F and TGX1835-10E in all the agro-ecologies. The TGX1951-3F is, therefore, recommended for optimum grain yield in the savannas of northern Nigeria. However, the late maturing variety TGX1904-6F is not recommended for the SS due to the short growing season in this zone.

## Introduction

The cultivation of soybean is increasing in the Nigeria savannas as a result of its importance as a major cash crop used in the food and feed industry^1,2^. Nigeria is the second largest producer of soybean in Africa after South Africa producing 630,000 Mt of the crop in 2019^3^. The crop provides opportunity to diversify the cereal cropping systems in the savannas of West Africa. Mixed with cereals, the resulting diet meets the standards of the United Nations Food and Agriculture Organization^4^. Soybean contains 20% oil that is 85% unsaturated and cholesterol free.

Farmers have adopted several new cultivars developed at the International Institute of Tropical Agriculture (IITA)^5^ and unlike cowpea the crop do not require chemical pest control. Soybean also nodulates freely with native rhizobia strains thereby taking care of their nitrogen (N) need through biological nitrogen fixation when the plants are well established^5^. In West Africa including Nigeria, smallholder farmers cultivate soybean mainly in the savanna belt under rainfed conditions^6,7^. Despite the availability of improved varieties, several factors limit the yield of soybean in the Nigeria savannas. Poor soil fertility^7,8^, drought caused by variability in rainfall, and diseases^9^ are the major constraints to soybean production in the Nigeria savannas. Soybean yields as low as less than 1 metric ton ha^−1^ are reported without the use of inputs and other crop management practices^9,10^. In Nigeria, soybean is cultivated predominantly in the Guinea savannas where rainfall is enough to support growth and development. Because of the availability of early maturing varieties, soybean production has spread to the Sudan savannas^2^ where rainfall amount is less and the growing season is shorter. In the Nigeria savannas, delays in onset of the rainy season are prevalent. The variations in the onset, amount and duration of rainfall are very high across years^11^. Also, intermittent droughts during the growing seasons are becoming frequent even in the high rainfall zones in the Guinea savannas^12^. Because soybean is very sensitive to drought stress^13^, sowing should be timed to avoid early season drought and drought at the end of the cropping season. In order to increase the production of crops including soybean in the Nigeria savannas, production practices should be properly designed to tolerate the low precipitation and high air temperatures that characterize the zone^14^. The use of adapted soybean varieties and optimum sowing windows are avenues to increase productivity in the face of climate variability. In the Guinea and Sudan savannas of Nigeria, the length of the growing season depends on the first rain, which is highly variable^12^. Farmers in the Nigeria savannas plant soybean between mid-June and first week of July depending on when the rains establish. It is necessary for producers to know the extent to which sowing can be delayed and the likely yield penalty they could experience as a result of late sowing.

In the Nigeria savannas the recommendations for sowing windows of annual crops are usually based on agronomic field experiments conducted in specific fields and regions^12^. Majority of such trials cannot be temporally and spatially replicated because of seasonal variations^15^. Determination of optimum sowing dates for crops involves several experiments covering large areas and repeated over long period of time to capture the seasonal variability in the onset, amount of rainfall and distribution^15^. Also, data for one location is not useful for another location because of variation in rainfall, temperature and soil conditions^16^. Several related factors such as location, year-to-year climate conditions, and maturity group selection make the determining of the optimum sowing date for soybeans a complex decision^17,18^. Our inability to control and manipulate environmental factors in the field makes it difficult to investigate their effects with traditional field experiments^19^.

Crop simulation models have been developed and used as tools for the evaluation of alternative management options for a particular location, including fertilizer application rates, plant density, sowing dates and land use^12,20–22^. Decision Support System for Agrotechnology Transfer (DSSAT) gives opportunities for extrapolating results from short-season field experiments to other years and locations with the use of soil information and long-term weather data^23^. The CROPGRO-Soybean model in DSSAT has been tested and evaluated extensively by many researchers across locations and found good correlations between observed and simulated values for a wide range of experimental practices against field data and environmental conditions around the world^24–28^. These include inorganic fertilizer management in Nigeria^12,29^, crop management strategies in extension systems in Kenya^30^, investigation of variety and sowing time technologies in Nigeria^14,15,31^ and Togo^32^, varietal performance of soybean in diverse ecologies in Kenya^25^.

CROPGRO-soybean was designed to mimic soybean behaviour and has been successfully tested under a wide range of environments^19,33,34^. Banterng et al.^26^ evaluated the performance of soybean varieties in Thailand under optimum management practices using the CROPGRO-Soybean model. They found out the model predicted growth and yield parameters that were in good agreement with the observed parameters. For maximum grain yield, the optimum sowing window of 15th June to 15th July was simulated under rainfed conditions in Thailand. Egli and Bruening^19^ investigated the effects of planting date on soybean yield using SOYGRO V5.41. The model accurately predicted observed yield responses for planting date from several locations in Kentucky, USA. Nyambane et al.^25^ simulated the phenology and yield of dual-purpose soybean varieties under different environments in Kenya using CROPGRO-Soybean model. The model predicted flowering, first pod and maturity dates within acceptable ranges for the Kitale site. The model reasonably predicted yield for the soybean varieties with low RMSEs ranging from 40 to less than 300 kg ha^−1^. Simulated grain yield ranged from 468 to 699 kg ha^−1^ in Msabaha, 932 to 1682 kg ha^−1^ in Kakamega and 1107 to 2617 kg ha^−1^ in Kitale sites. To our knowledge the performance of the CROPGRO-Soybean model has not been evaluated for prediction of soybean growth and yield in the Nigeria savannas. The objectives of this study were to evaluate the ability of CSM-CROPGRO-Soybean model to simulate growth, development and yield of contrasting soybean varieties and to determine optimum sowing windows for the soybean varieties for diverse growing environments in the savannas of Nigeria.

## Materials and methods

### Experiments for model calibration and evaluation

Experiments were conducted to collect data for the calibration and evaluation of the CROPGRO-Soybean model. For model calibration, we conducted 10 experiments across two sites in the Sudan savannas of Nigeria. The experiments were conducted in the rainy seasons at the Bayero University, Kano (BUK) Agricultural Research Farm (11° 59′ N; 8° 25′ E; 466 m a.s.l) and Audu Bako College of Agriculture, Dambatta (12° 19′ N; 8° 31′ E; 504 m a.s.l). In the two locations, the experiments were conducted in 2016, 2017, 2018 and 2019. In 2018, two experiments were conducted on different dates at both sites. At BUK, planting was done on 13th June in 2016, 3rd July in 2017, 27th June and 10th July in 2018, and 6th July in 2019. At Dambatta, sowing was done on 22nd June in 2016, 1st July in 2017, 26th June and 9th July in 2018 and 4th July in 2019. In each experiment, three soybean varieties that have been released in the Nigeria savannas were laid out as a single factor experiment in a randomized complete block design with three replications. The experiments were conducted under optimum management practices with no water and nutrients stress. Prior to land preparation, 4 tonnes ha^−1^ of poultry manure was applied to the plots to increase soil fertility. The experimental field was disc-harrowed and ridged before sowing. Each plot contains four 5 m-long rows with spacing of 0.75 m between rows. Each plot received a basal application of 40 kg ha^−1^ each for K_2_O in the form of Muriate of Potash and P_2_O_5_ in the form of Triple Super Phosphate (TSP). Before sowing, a Nodumax inoculant containing rhizobia was used to inoculate the seeds. Seven seeds were planted at intra-row spacing of 10 cm, and later thinned to four plants per hill to give a final population of 533,333 plants ha^−1^. Although the experiments were conducted in the rainy season, supplementary irrigation was carried out when necessary to keep the plots well-watered.

The experiments for model evaluation were conducted at the International Institute of Tropical Agriculture (IITA) experimental stations at Zaria (11° 11′ N, 7° 38′ E, 686 m a.s.l) and Doguwa (10° 45′ N, 8° 44′ E, 466 m a.s.l) in the northern Guinea savanna (NGS) zone. The experimental fields were disc-harrowed and ridged. The sowing was done on 20th and 24th June in 2016 at Zaria and Doguwa, respectively and 7th July in 2017 and 22nd June in 2018 at Zaria only. The experiments were design using split-plot design with three replications. The main plot treatments consisted of three P fertilization rates (0, 20, and 40 kg P_2_O_5_ ha^−1^). The subplot treatments were three soybean varieties (TGX 1835-10E, TGX1951-3F and TGX 1904-6F). The sub-plot size was 3 × 5 m with four 5-m rows spaced at 0.75 m. The harvest area was 1.5 × 4.5 m. Soybean was planted at the same plant population as for model calibration. All the experiments were carried out in compliance with relevant guidelines and regulations.

### Weather and soil data

WatchDog weather station (manufactured by Spectrum Technologies Inc, USA) was used to monitor daily weather conditions at experimental sites. The stations monitored daily rainfall, minimum and maximum air temperature, and solar radiation used for model calibration and evaluation. For the long-term simulation of soybean response to sowing window, a 30-year weather data (1985–2014) was obtained from the Nigeria Meteorological Agency (NIMET) for Kano representing Sudan savanna (SS) and Zaria representing northern Guinea savanna (NGS). For Jagiri representing southern Guinea savanna (SGS), thirty years’ records of daily precipitation were sourced from downscaled CHIRPS rainfall at 5.5 km resolution^35^, then merged with data (daily minimum and maximum air temperatures, and daily solar radiation) from National Aeronautics and Space Administration (NASA) database for Climatology Resource for Agro-climatology (http://power.larc.nasa.gov/). R scripts were created to append data for CHIRPS and NASA power. The weatherman utility software in the DSSAT v4.7 was used to input the weather data for the 2 sites in order to check for errors before use.

Before the trials were established, a detailed soil characterization and profiling was done at each experimental site and the long-term simulation sites. FAO guidelines were used to classify the generic horizons of the soil profile and types^36^. The modified Walkley and Black chromic acid wet chemical oxidation and spectrophotometric method was used to measure total soil organic carbon (total OC)^37^. Total nitrogen (total N) was determined by the micro-Kjeldahl digestion method^38^. Soil pH in water (S/W ratio of 1:2.5) was measured using a glass electrode pH meter and followed by the particle size distribution using the hydrometer method^39^. Available phosphorus was extracted using the Bray 1 method^40^. Phosphorus in the extract was determined colorimetrically by the molydo-phosphoric blue method using ascorbic acid as a reducing agent. K was analysed based on Mehlich 3 extraction procedure^41^. The soil utility software (SBuild) of DSSAT was used to input the soil profile information. The P soil test information was entered into the model in the soil analysis layer and the P balance turned on in the simulation option. For each soil layer, the model estimated bulk density, drained upper limit (DUL), saturated upper limit (SAT), lower limit of plant-available soil water (LL), saturated hydraulic conductivity (Ksat) and root growth factor (RF) using the soil data tool SBuild in DSSAT. The Soil parameters used in the model is presented in Table 1.

**Table 1:** Soil physical and chemical properties of pedons used for calibration, evaluation and model applications in the Sudan savanna and northern Guinea savanna of Nigeria.

Depth (cm)	LL (mm/mm)	DUL (mm/mm)	SAT (mm/mm)	BD (g/cm^3^)	OC (g kg^−1^)	Sand (%)	Silt (%)	Clay (%)	pH (1:2.5 H_2_0)	N (g kg^−1^)	Meh P (mg kg^−1^)
**BUK-Kano**											
0–28	0.100	0.201	0.401	1.56	4.4	68	12	20	6.6	0.4	5.6
28–58	0.127	0.207	0.382	1.58	2.2	65	16	19	6.7	0.2	0.7
58–120	0.112	0.194	0.385	1.57	2.1	66	18	16	5.9	0.2	0.2
120–156	0.112	0.191	0.376	1.60	0.4	65	18	17	7.0	0.2	0.1
156–210	0.102	0.180	0.376	1.60	0.4	67	18	15	6.1	0.2	0.7
**Danbatta**											
0–22	0.106	0.167	0.366	1.62	3.6	80	6	14	5.7	0.3	2.7
22–49	0.105	0.155	0.350	1.67	1.4	82	3	15	6.3	0.2	3.0
49–69	0.110	0.162	0.349	1.67	2.0	82	2	16	6.0	0.2	1.0
69–116	0.110	0.158	0.346	1.68	1.4	82	2	16	5.8	0.1	0.7
116–190	0.117	0.169	0.347	1.68	0.5	78	4	18	5.8	0.2	0.4
**Doguwa**											
0–10	0.139	0.277	0.452	1.38	4.0	31	28	41	4.8	0.11	6.1
30-Oct	0.281	0.410	0.467	1.34	1.6	19	24	57	4.8	0.07	3.0
30–74	0.281	0.394	0.450	1.39	1.1	13	24	63	4.9	0.04	2.8
74–106	0.260	0.375	0.443	1.41	0.9	23	20	57	5.8	0.04	2.6
**Zaria**											
0–20	0.139	0.277	0.452	1.38	4.6	38	42	20	4.7	0.8	1.9
20–45	0.281	0.410	0.467	1.34	4.1	21	30	49	5.6	0.7	3.0
45–84	0.281	0.394	0.450	1.39	1.7	26	24	50	5.7	0.2	2.8
84–120	0.260	0.375	0.443	1.41	3.2	28	26	46	5.2	0.2	2.8
120–190	0.267	0.377	0.441	1.42	2.7	28	24	48	5.0	0.1	1.3
**Jagiri**											
0–23	0.063	0.152	0.461	1.37	5.4	71.7	10.7	17.6	5.7	0.7	7.01
23–65	0.089	0.174	0.354	1.51	1.6	29.7	8.7	61.6	5.2	0.4	4.29
65–97	0.131	0.212	0.447	1.37	3.2	33.7	14.7	51.6	5.5	0.4	5.85
97–156	0.120	0.211	0.443	1.62	1.6	43.7	14.7	41.6	5.5	0.4	6.04

*LL* lower limit, *DUL* drained upper limit, *SAT* volumetric water content at saturation, *BD* bulk density, *OC* organic carbon content, *N* percent Nitrogen content, *P* available phosphorus.

### Plant measurements

Field data were collected from the net plot leaving the outside rows and a distance of 25 cm at the end of each row to serve as borders. Parameters measured include phenology dates, total dry matter m^−2^, and grain yield (kg ha^−1^). At full podding, a quadrat measuring 1 m × 1.5 m was placed across the two middle rows of the net plot; all the plants in the quadrat were sampled to determine dry matter. Leaves were collected from the quadrat on a daily basis until harvest. At harvest, all plants from the quadrat were removed and separated into pods, stem and leaves. The pods that contain seeds were threshed and separated from seeds. All plant parts including leaves collected from the quadrat were dried in an oven at 60 °C for 76 h to constant weight, weighed and combined to give a total dry matter. Grain yield was determined according to Kamara et al.^42^. Pods on plants from the two middle rows were harvested, sundried, and hand threshed. The grains obtained from the quadrat were added to those from the two middle rows and weighed to calculate grain yield. The moisture content of grain samples from each plot was determined using portable moisture meter (Farmex MT-16). Grain yield kg ha^−1^ was calculated based on 13% moisture content.

### Crop model overview

DSSAT shell allows the users to organize and manipulate crop, soil and weather data, to run crop models in various ways, and to analyze their outputs^43,44^. CROPGRO-Soybean which is one of the models running under DSSAT, is a generic physiological process-oriented legume crop model^45^. The CSM-CROPGRO-Soybean model within the DSSAT suite simulates plant growth and development from sowing to maturity using a daily time step, and ultimately predicts yield^24,26,46^. The basic structure of the model, including underlying equations, has been published by several authors^47–49^. The model accounts for vegetative and reproductive development; photosynthesis, respiration and partitioning; and transpiration, root water uptake, soil evaporation, soil water flow, infiltration and drainage^45^. The soil, weather, and crop management information are the minimum data sets required to run the model. The simulated physiological processes characterize the crop’s response to the major weather factors, such as temperature, rainfall and solar radiation, and to soil characterizations such as the amount of extractable soil water and nutrients^26^. The CROPGRO-Soybean model includes detailed soil and plant nitrogen balance components which simulate nitrogen uptake, nitrogen fixation and nitrogen mobilisation^49^.

### Methods of model calibration and evaluation

Model calibration is the adjustment of certain model parameters to make the model work for any desired location. The CROPGRO-Soybean model requires genetic coefficients that describe durations of phases of the crop life cycle for vegetative growth and reproductive traits unique to a given cultivar^50^. These coefficients allow the model to predict differences in development, growth and yield among cultivars when planted in the same environment as well as the differences in the behaviour of a single cultivar when planted in different environments^27^. The soybean model was calibrated by determining the cultivar coefficients for the three cultivars TGX 1835-10E, TGX1951-3F, and TGX 1904-6F using the experimental data. Fifteen cultivar coefficients are required by the CROPGRO-Soybean model. The cultivar coefficient describes the growth and development of the crop. As these were not available for the cultivars used in these experiments, the existing cultivar coefficients for the maturity group (MG) 7 were used for the cultivar TGX 1835-10E; while Jupiter 10 was used for the other cultivars at the start of the calibration to represent the characteristics of a tropical soybean varieties.

The cultivar coefficients for each cultivar were determined through trial and error of the model and by comparing simulated and observed data, following the procedures described by Hoogenboom et al.^51^. To obtain the critical short-day length (CSDL) hour, and photoperiod sensitivity (PPSEN) 1/h, the simulated annealing was used by matching the simulated and observed flowering date. The cultivar coefficients EMFL (the duration from emergence to flowering) photo thermal days (Pd), FLSH (flowering to beginning pod) Pd, FLSD (flowering to beginning seed) Pd and SDPM (beginning seed to physiological maturity) Pd, were adjusted for the simulated and observed data to match the life cycle of the crop. The value for maximum leaf photosynthesis rate (LFMAX) mg CO_2_/m^2^/s, was modified using trial and error to obtain a good agreement between simulated and observed dry matter accumulations. The specific leaf area coefficient (SLAVR) cm^2^/g, the time to cessation of leaf expansion (FLLF) Pd, and maximum size of full leaf (SIZLF) cm^2^, were adjusted to minimize the difference between the observed and simulated leaf growth. The average number of seeds per pod (SDPDV) #/pod, duration of pod addition (PODUR) Pd, maximum weight per seed (WTPSD) g, and seed filling duration for a pod cohort (SFDUR) Pd, were also adjusted to match the simulated and observed pod weight.

The performance of the model was evaluated with the four independent data sets from the P response trials as described in 2.1. Model performance was evaluated using statistical indicators of root mean square error (RMSE) and the index of agreement (d value)^52^ between observed and simulated variables. Data used for evaluation included days to flowering and maturity and final grain yield.

### Statistical evaluation of model performance

The model statistics used to evaluate model performance including RMSE, normalize RMSE (nRMSE) and *d* statistic^12,15,53^.

$$\mathrm{RMSE}={\frac{\sqrt{1}}{\mathrm{N}}\sum \left(\mathrm{Oi}-\mathrm{Pi}\right)2},$$

P is the predicted, O is the observe, N is the number of observations within each treatment.

$$\mathrm{nRMSE}= \frac{\mathrm{RMSE x }100}{\mathrm{\grave{O} }},$$

$\mathrm{\grave{O} }$ is the overall mean of observed values

$$d = 1 - \frac{{\sum _{{i = 1}}^{n} (m_{i} - S_{i} )^{2} }}{{\sum _{{i = 1}}^{n} |S_{\grave{i}} | + |m_{\grave{i}} |)^{2} }},$$

$$S_{\grave{i}} = S_{i} - \bar{m}$$ and $${m}_{\grave{i}}$$ = $${m}_{i}-\overline{m }.$$

The simulation is considered excellent when the nRMSE is < 10%, good if the nRMSE is > 10% and < 20%, fair if the nRMSE is > 20% and < 30%, and poor if the nRMSE is > 30%^54^. The *d* statistic is recommended for making cross-comparisons when the *d* value is both relative and has bounded measures^52^.

### Long-term seasonal analyses

Seasonal analysis was carried out under rainfed-conditions to test the effect of varying sowing windows on yield of soybean in three diverse locations in the Nigeria savannas. Kano (11.516° N, 8.516° E, 466 m a.s.l) in the Sudan savanna, Zaria (11.054° N, 7.702° E, 686 m a.s.l) in the northern Guinea savanna, and Jagiri (9.417° N, 8.117° E, 400 m a.s.l) in the southern Guinea savanna zones of Nigeria. The simulation was applied over a 30-year period (1985–2015) at varying sowing windows using the daily rainfall, temperature and solar radiation and soil information obtained from the three locations. The model was set to consider ten sowing windows (SWs) implemented in CROPGRO-Soybean Model: June 1–7 (SW1), June 8–14 (SW2), June 15–21 (SW3), June 22–28 (SW4), June 29–July 5 (SW5), July 6–12 (SW6), July 13–19 (SW7), July 20–26 (SW8), July 27–August 2 (SW9) and (SW10) August 3–9. Sowing was carried out based on the treatments with conditions set to sow when a total rainfall is above 10 mm within the last three days before sowing in each simulation year. Generally, the sowing density was 53.3 plants m^−2^, planted at a soil depth of 4 cm. The model was set to supply 40 kg P_2_O_5_ at sowing using TSP fertilizer material. The model was set to harvest at harvest maturity. The standard deviations, the mean maximum and minimum yields for 30 years were calculated for each variety and location. The results of simulated grain yield over 30-year period were presented using cumulative frequency plots.

## Results

### Soil and weather

The results of soil chemical and physical properties for model calibration, evaluation, and application are shown in Table 1. Soil data from the study sites showed wide variability in major properties on the top layer, with soil pH ranging from 4.7 at Zaria to 6.6 at Kano, and the available phosphorus from 2.7 at Kano to 7.0 mg kg^−1^ at Jagiri. Soil total nitrogen (total N) content was very low across the study areas, ranging from 0.1 g kg^−1^ at Doguwa to 0.8 g kg^−1^ at Zaria. All sites, except at Jagiri had low available P below the critical value for soybean of 7 mg kg^−1^^55^. Soils in Dambatta, Jagiri and Kano were sandy, with a sand content of above 67% in the top layer, whereas Doguwa and Zaria were loamy soils with a low sand content of below 40%. Soil organic carbon was < 1% (10 g kg^−1^) in all the study sites and ranged from 3.6 g kg^−1^ at Dambatta to 5.4 g kg^−1^ at Jagiri. The average bulk density for all the study sites was below 1.6 g cm^−3^ which was best for root growth and aeration^41^. The soil hydraulic parameters (LL, DUL and SAT) showed high variability among the various soil layers in the study areas (Table 1).

For model calibration sites, rainfall was 763, 641, 575 and 592 mm in 2016, 2017, 2018 and 2019, respectively at BUK. In Dambatta rainfall was 794, 745, 646 and 606 mm in 2016, 2017, 2018 and 2019, respectively. In Kano, the minimum and maximum air temperatures recorded during the experimental period were 19.7 and 34.3 °C in 2016, 23.9 and 29.4 °C in 2017, 20.8 and 33.5 °C in 2018 and 20.5 and 32.8 °C in 2019, respectively. At Dambatta, the minimum and maximum air temperatures were 19.1 °C and 35.1 °C in 2016, 20.1 °C and 35.0 °C in 2017 °C, 18.4 °C and 34.8 °C in 2018 and 18.9 and 33.9 °C in 2019, respectively. During model evaluation experiment, the total annual rainfall in Doguwa was 1760 mm in 2016. The average minimum and maximum air temperatures were 19.8 and 32.3 °C. In Zaria, the total annual rainfall during the experimental period was 875 mm in 2017 and 777 mm in 2018. The minimum and maximum air temperatures were 19.9 and 20.8 °C in 2016, 18.5 and 32.4 °C in 2017 and 18.7 and 32.3 °C in 2018, respectively.

Result from long-term weather data of 30-years (1985–2014) monthly variations of rainfall, rainy days (RD), minimum and maximum air temperatures are shown in Table 2. The results revealed that rainfall increases southwards with a mono-modal pattern indicating a short growing season (May–Oct) for Kano and Zaria while Jagiri indicates growing season between April and October.

**Table 2:** Long-term (1985–2014) Monthly variation of rainfall, number of rainy days (NRD), minimum temperature (Tmin) and maximum temperature (Tmax) in Kano (Sudan Savanna), Zaria (northern Guinea savanna) and Jagiri (southern Guinea savanna).

Month	Kano				Zaria				Jagiri			
	Rainfall	NRD	T_min_	T_max_	Rainfall	NRD	T_min_	T_max_	Rainfall	NRD	T_min_	T_max_
	(mm)		(°C)		(mm)		(°C)		(mm)		(°C)	
Jan	0	0	14.3	30.4	0	0	14.8	30.8	0	0	17.1	33.4
Feb	0	0	16.9	33.0	0	0	17.3	33.4	4	2	19.4	35.5
Mar	1	0	21.0	37.2	4	0	20.7	36.1	29	6	21.7	36.0
Apr	7	2	24.6	39.3	36	3	22.9	36.0	83	17	22.4	33.7
May	52	5	25.0	38.1	119	9	22.5	33.3	155	15	22.0	30.6
**Jun**	**123**	**9**	**23.5**	**34.7**	**144**	**11**	**21.4**	**30.5**	**179**	**21**	**21.1**	**28.6**
**Jul**	**214**	**14**	**21.9**	**31.8**	**226**	**14**	**20.7**	**28.9**	**245**	**21**	**20.5**	**27.4**
**Aug**	**267**	**15**	**21.4**	**31**	**288**	**17**	**20.5**	**28.5**	**350**	**21**	**20.3**	**26.7**
Sep	117	9	21.8	32.8	178	13	20.7	30.1	271	21	20.3	27.6
Oct	15	1	20.7	34.6	48	4	19.3	32.1	139	16	20.1	28.9
Nov	0	0	16.5	33.5	0	0	16.0	33.1	4	1	17.9	31.0
Dec	0	0	14.2	30.5	0	0	14.9	31.2	0	0	16.5	32.0
Total/mean	795	55.3	20.1	33.9	1042	71	19.3	32	1462	140	20.0	30.9
CV (%)	20.2	15.3	7.9	5.2	14	8.8	8.5	5.82	8.0	7.3	4.4	4.6

The bold represents sowing months used for the simulation.

### Model calibration

**Figure 1:**
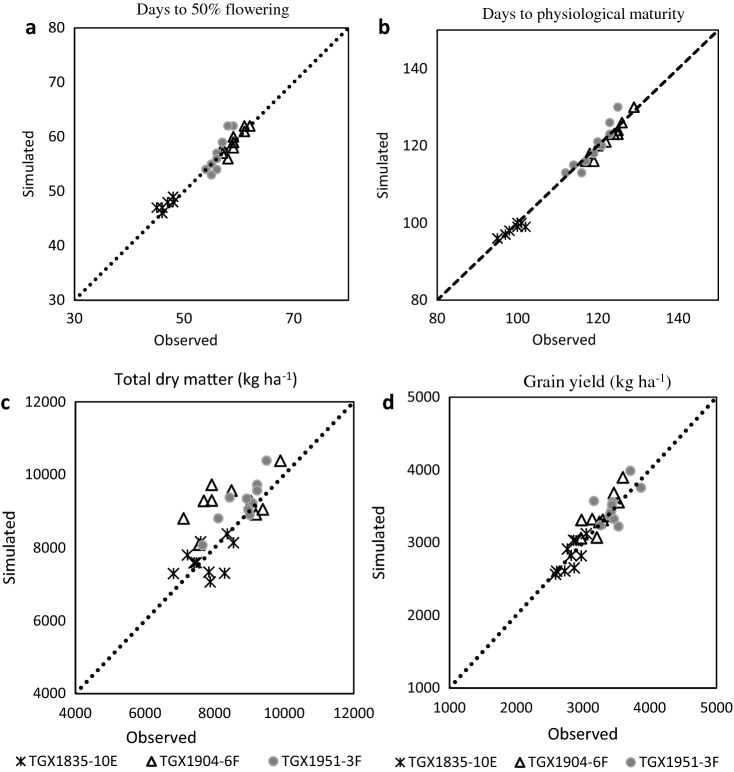
(**a**) Observed vs. simulated days to 50% flowering using calibration experiment conducted 2016–2019 growing seasons for cultivar TGX1835-10E (RMSE = 0.95  kg ha^−1^, RMSE_n_ = 2.0%, D = 0.8); TGX1904-6F (RMSE = 0.89 kg ha^−1^, RMSE_n_ = 1.5%, D = 0.93); TGX1951-3F (RMSE = 1.95 kg ha^−1^, RMSE_n_ = 3.5%, D = 0.83). (**b**) Observed vs. simulated days to days to physiological maturity using calibration experiment conducted 2016–2019 growing seasons for cultivar TGX1835-10E (RMSE = 1.18 kg ha^−1^, RMSE_n_ = 1.2%, D = 0.9); TGX1904-6F (RMSE = 1.34 kg ha^−1^, RMSE_n_ = 1.1%, D = 0.97); TGX1951-3F (RMSE = 2.21 kg ha^−1^, RMSE_n_ = 1.9%, D = 0.94). (**c**) Observed vs. simulated total dry matter using calibration experiment conducted 2016–2019 growing seasons for cultivar TGX1835-10E (RMSE = 545.42 kg ha^−1^, RMSE_n_ = 7.1%, D = 0.63); TGX1904-6F (RMSE = 1111.03 kg ha^−1^, RMSE_n_ = 13.3%, D = 0.6); TGX1951-3F (RMSE = 538.46 kg ha^−1^, RMSE_n_ = 6.1%, D = 0.8). (**d**) Observed vs. simulated grain yield using calibration experiment conducted 2016–2019 growing seasons for cultivar TGX1835-10E (RMSE = 125.84 kg ha^−1^, RMSE_n_ = 4.5%, D = 0.85); TGX1904-6F (RMSE = 177.44 kg ha^−1^, RMSE_n_ = 5.4%, D = 0.85); TGX1951-3F (RMSE = 197.74 kg ha^−1^, RMSE_n_ = 5.7%, D = 0.75).

Table 3 shows the values of calibrated parameters for the three soybean cultivars. The cultivar coefficients for the soybean cultivars TGX1951-3F, TGX1904-6F and TGX1835-10E were estimated through trial and error and comparison of model simulated and observed values. The CROPGRO-Soybean model adequately simulated all the measured parameters of all the varieties using the generated cultivar coefficients (Fig. 1).

**Table 3:** Genetic coefficients of soybean cultivars used in the study.

Coefficient	Definition	Unit	TGX1835-10E	TGX1951-3F	TGX1904-6F
CSDL	Critical short-day length below which reproductive development progresses with no day-length effect (for short-day plants)	h	11.88	11.37	11.25
PPSEN	Slope of the relative response of development to photoperiod with time (positive for short-day plants)	1/h	0.311	0.340	0.312
EM-FL	Time between plant emergence and flower appearance (R1)	pd^*^	29.94	27.84	29.94
FL-SH	Time between first flower and first pod (R3)	pd	7.000	6.000	6.000
FL-SD	Time between first flower and first seed (R5)	pd	20.85	14.35	14.40
SD-PM	Time between first seed (R5) and physiological maturity (R7)	pd	15.35	21.35	21.00
FL-LF	Time between first flower (R1) and of leaf expansion	pd	15.00	15.00	18.00
LFMAX	Maximum leaf photosynthesis rate at 30 0C, 350 vpm CO_2_, and high light	mg CO_2_/m^2^/s	1.016	1.016	1.016
SLAVR	Specific leaf area of cultivar under standard growth conditions	cm^2^/g	315.3	315.3	315.3
SIZLF	Maximum size of full leaf (three leaflets)	cm^2^	220.6	230.6	220.6
WTPSD	Maximum weight per seed	g	0.184	0.184	0.184
SFDUR	Seed filling duration for pod cohort at standard growth conditions	pd	20.27	18.45	25.27
SDPDV	Average seed per pod under standard growing conditions	#/pod	2.090	2.090	2.090
PODUR	Time required for cultivar to reach final pod load under optimal conditions	pd	10.00	10.00	10.00

^*^pd photothermal days.

The average predicted days to flowering, days to physiological maturity and grain yield of soybean varieties range from 47 to 59 days, 99 to 122 days and 2815 kg ha^−1^ to 3496 kg ha^−1^ respectively (Fig. 1). The values of RMSE and NRMSE ranged from 0.89 to 1.95 days and 1.5 to 3.5% for days to flowering, respectively. The values of RMSE and NRMSE ranged from 1.18 to 2.21 days and 1.1 to 1.9% for days to physiological maturity, respectively. For grain yield the RMSE values ranged from 126 to 198 kg ha^−1^ and NRMSE (%) ranged from 4.5 to 5.7%. The d-index values for days to 50% flowering ranged from 0.8 to 0.93, days to physiological maturity 0.9–0.97, total dry matter 0.6–0.8 and grain yield 0.75–0.85 for all the varieties (Fig. 1). The statistical indices for calibration indicated good agreements between observed and simulated values for all measured parameters for the three soybean varieties.

### Model evaluation

The accuracy of the CROPGRO-Soybean model simulations and performance of genetic coefficients were assessed by running the model with four independent data sets collected during the 2016 cropping season at Doguwa and, 2016, 2017 and 2018 cropping seasons at Zaria for three levels of P (0, 20 and 40 kg P_2_O_5_ ha^−1^) application. Days to 50% flowering, days to physiological maturity and grain yield were used for model evaluation. The simulated and observed values with their predictive difference are presented in Table 4. Except for grain yield with variety TGX1904-6F, the model simulated days to 50% flowering, physiological maturity and grain yield reasonably well across P levels as indicated by low nRMSE (< 20%) and high *d-index* values (0.73–0.99) for the three varieties in the two locations. The values of RMSE were low < 2 days for days to 50% flowering and physiological maturity and < 300 kg ha^−1^ for grain yield for all the varieties in both locations.

**Table 4:** Simulated (S) and observed (O) days to 50% flowering, physiological maturity and grain yield obtained from evaluation experiments conducted at two locations in Doguwa and Zaria.

Location	Variety	P rate (kg P_2_O_5_ ha^−1^)	Days to 50% flowering			Days to physiological maturity			Grain yield (kg ha^−1^)		
			S	O	S–O	S	O	S–O	S	O	S–O
Doguwa	TGX1835-10E	0	46	44	2	95	94	1	375	224	151
		20	46	46	0	95	96	− 1	1606	1435	171
		40	46	46	0	95	96	− 1	1879	1713	166
Zaria		0	48	46	2	97	97	0	175	109	66
		20	48	48	0	98	99	− 1	1350	1160	190
		40	48	48	0	98	99	− 1	1440	1410	30
RMSE					1.26			1.04			148.7
NRMSE (%)					2.70			1.10			15.6
*d* value					0.73			0.94			0.99
Doguwa	TGX1951-3F	0	53	50	3	112	110	2	477	528	− 51
		20	53	54	− 1	112	114	− 2	1409	1379	30
		40	53	54	− 1	113	112	1	2113	2089	24
Zaria		0	57	54	3	116	114	2	173	64	109
		20	57	56	1	113	115	− 2	1095	1126	− 31
		40	57	56	1	114	115	− 1	1655	1858	− 203
RMSE					1.91			1.71			172.3
NRMSE (%)					3.5			1.5			15.7
*d* value					0.8			0.92			0.98
Doguwa	TGX1904-6F	0	53	50	3	112	110	2	436	535	− 99
		20	53	54	− 1	112	114	− 2	1549	1620	− 71
		40	53	54	− 1	113	112	1	2016	1989	27
Zaria		0	57	54	3	116	114	2	160	78	82
		20	57	56	1	113	115	− 2	1250	1344	− 94
		40	57	56	1	114	115	− 1	1497	1755	− 258
RMSE					1.91			1.71			272.9
NRMSE (%)					3.4			1.5			23.9
*d* value					0.8			0.92			0.96

*d*-Willmott index of agreement, (Willmott, 1982) ranging from 0 to 1, 1 being perfect agreement.

### Seasonal analysis of soybean grown under different sowing windows

The simulated grain yields for the ten (10) sowing windows (SWs) using 30-year weather data (1985–2014) for the three soybean varieties are presented in Tables 5, 6, 7. Generally, the simulation outputs revealed that soybean varieties respond to varying SWs and sites across the agro-ecological zones. The results showed higher grain yields for the soybean varieties in northern Guinea than in southern Guinea and Sudan savanna agro-ecological zones. The least grain yields were simulated in the southern Guinea savanna despite higher rainfall than for the other zones. In addition, higher yield was simulated for the variety TGX1951-3F than for TGX1904-6F and TGX1835-10E.

**Table 5:** Simulated grain yield (kg ha^−1^) of different sowing windows based on 30‐year (1985–2014) seasonal analysis for soybean varieties (TGX1835‐10E, TGX1904-6F and TGX1951‐3F) in Kano (Sudan savanna).

Sowing window	TGX1835-10E				TGX1904-6F				TGX1951-3F			
	Mean	St. Dev	Max	Min	Mean	St. Dev	Max	Min	Mean	St. Dev	Max	Min
SW1	1763	259	2127	986	1590	221	1935	1115	1714	218	2049	1229
SW2	1635	222	2099	1205	1475	248	1852	767	1613	253	1996	898
SW3	1631	207	1960	1078	1399	268	1850	652	1542	267	2005	804
SW4	1534	275	1917	859	1261	297	1794	552	1411	310	1966	646
SW5	1366	286	1901	558	1109	331	1699	441	1266	347	1855	554
SW6	1195	350	1861	458	911	329	1558	330	1064	347	1758	432
SW7	981	357	1684	315	731	305	1320	201	874	329	1510	279
SW8	787	362	1476	218	574	260	1077	109	706	309	1265	176
SW9	595	294	1139	135	458	209	871	75	569	260	1051	107
SW10	453	271	1068	117	383	164	760	94	474	207	922	146

SW1: June 1–7; SW2: June 8–14; SW3: June 15–June 21; SW4: June 22–28 June; SW5: June 29–July 5; SW6: July 6–July 12; SW7: July 13–July 19; SW8: July 20–July 26; SW9: July 27–August 2; SW10: August 3–August 9.

**Table 6:** Simulated grain yield (kg ha^−1^) of different sowing windows based on 30‐year (1985–2014) seasonal analysis for soybean varieties (TGX1835‐10E, TGX1904-6F and TGX1951‐3F) in Zaria (northern Guinea savanna).

Sowing window	TGX1835-10E				TGX1904-6F				TGX1951-3F			
	Mean	St. Dev	Max	Min	Mean	St. Dev	Max	Min	Mean	St. Dev	Max	Min
SW1	1632	165	1995	1351	1858	124	2031	1452	1968	121	2162	1596
SW2	1670	170	2054	1342	1867	136	2010	1383	1992	147	2168	1470
SW3	1610	185	1946	1215	1821	164	2061	1216	1959	166	2229	1394
SW4	1571	232	2003	970	1743	206	1992	990	1886	215	2162	1120
SW5	1497	206	1826	964	1657	257	1913	743	1809	256	2119	876
SW6	1403	209	1766	798	1545	278	1864	630	1701	274	1990	741
SW7	1264	228	1627	571	1396	325	1959	513	1570	326	2002	620
SW8	1162	229	1541	431	1229	310	1719	471	1401	319	1905	531
SW9	1075	246	1486	328	1076	295	1652	457	1251	319	1803	495
SW10	934	243	1307	232	912	258	1564	426	1077	288	1634	438

SW1: June 1–7; SW2: June 8–14; SW3: June 15–June 21; SW4: June 22–28 June; SW5: June 29–July 5; SW6: July 6–July 12; SW7: July 13–July 19; SW8: July 20–July 26; SW9: July 27–August 2; SW10: August 3–August 9.

**Table 7:** Simulated grain yield (kg ha^−1^) of different sowing windows based on 30‐year (1985–2014) seasonal analysis for soybean varieties (TGX1835‐10E, TGX1904-6F and TGX1951‐3F) in Jagiri (southern Guinea savanna).

Sowing window	TGX1835-10E				TGX1904-6F				TGX1951-3F			
	Mean	St. Dev	Max	Min	Mean	St. Dev	Max	Min	Mean	St. Dev	Max	Min
SW1	1384	124	1573	1026	1447	119	1674	1154	1531	124	1761	1189
SW2	1348	145	1561	932	1434	111	1646	1150	1527	117	1699	1189
SW3	1282	139	1510	902	1401	99	1646	1191	1492	104	1706	1244
SW4	1210	153	1419	769	1359	93	1561	1189	1455	104	1667	1214
SW5	1117	166	1379	708	1329	98	1518	1123	1414	103	1636	1210
SW6	1032	155	1251	681	1293	106	1440	1045	1386	112	1583	1107
SW7	986	146	1324	678	1262	120	1454	1023	1364	131	1561	1122
SW8	883	134	1098	550	1190	152	1460	875	1300	160	1609	1018
SW9	782	169	1213	434	1070	199	1415	675	1179	198	1528	790
SW10	725	147	1087	396	931	230	1283	529	1060	248	1448	640

SW1: June 1–7; SW2: June 8–14; SW3: June 15–June 21; SW4: June 22–28 June; SW5: June 29–July 5; SW6: July 6–July 12; SW7: July 13–July 19; SW8: July 20–July 26; SW9: July 27–August 2; SW10: August 3–August 9.

In Kano representing Sudan savanna (Table 5), simulated mean grain yields ranged from 453 to 1763 kg ha^−1^ for TGX1835-10E, 383–1590 kg ha^−1^ for TGX1904-6F and 474–1714 kg ha^−1^ for TGX1951-3F depending upon the sowing window (SWs). The result revealed that simulated yield decreases with delay in sowing. The mean simulated grain yield for sowing between SW1 and SW6 was above 1000 kg ha^−1^ except for TGX1904-6F (SW1–SW5) compared to SW7–SW10 for which the simulated yield was below 1000 kg ha^−1^. The most suitable SWs for stable grain yield were SW1 to SW5 for TGX1835-10E beyond which mean simulated yield declined by 13–67%. For TGX1904-6F and TGX1951-3F stable grain yields were simulated for SW1–SW4 beyond which yield decline significantly by 12–70% (TGX1904-6F) and 10–66% (TGX1951-3F) respectively.

In northern Guinea savanna, represented by Zaria (Table 6), the mean predicted grain yield ranged from 934 to 1670 kg ha^−1^ for TGX1835-10E, 912–1867 kg ha^−1^ for TGX1904-6F and 1077–1992 kg ha^−1^ for TGX1951-3F depending on the SWs. The highest yield was predicted for SW2 (8–14 June) and the lowest yield was simulated for SW10 (3–9 August). The result indicated that the most suitable SWs was June 1 to July 12 (SW1–SW6), beyond which, the predicted yield decline by 10–33% for TGX1835-10E, 10–41% for TGX1904-6F and 8–37% for TGX1951-3F. In the southern Guinea savanna represented by Jagiri site (Table 7), the yield ranges from 725 to 1384 kg ha^−1^ for TGX1835-10E, 931–1447 kg ha^−1^ for TGX1904-6F and 1060–1531 kg ha^−1^ for TGX1951-3F. Averaged simulated grain yield decrease with delay in sowing with highest yield simulated for SW1 and lowest yield for SW10 for all the tested varieties. The results show that the most suitable sowing windows for TGX1835-10E, TGX1904-6F, and TGX1951-3F were SW1–SW5, SW1–SW8 and SW1–SW9 beyond which yields will decline by 8–32%, 10–19%, and 10% for TGX1835-10E, TGX1904-6F, and TGX1951-3F, respectively.

**Figure 2:**
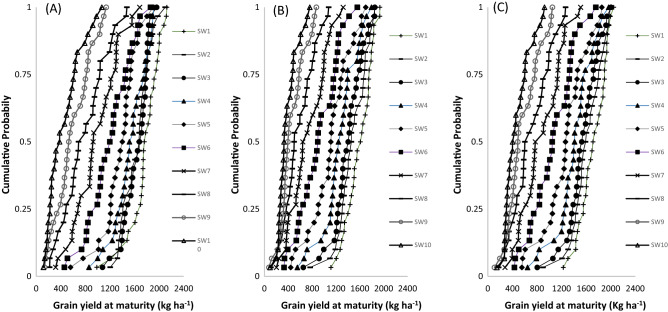
Cumulative function plots for simulated grain yields of TGX1835‐10E (**A**) TGX1904-6F (**B**) and TGX1951-3F (**C**) in Kano (Sudan savanna) over a 30‐year (1985–2014) period. SW1: June 1–7; SW2: June 8–14; SW3: June 15–June 21; SW4: June 22–28 June; SW5: June 29–July 5; SW6: July 6–July 12; SW7: July 13–July 19; SW8: July 20–July 26; SW9: July 27–August 2; SW10: August 3–August 9.

**Figure 3:**
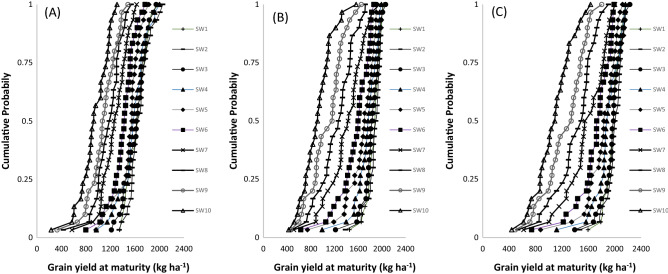
Cumulative function plots for simulated grain yields of TGX1835‐10E (**A**) TGX1904-6F (**B**) and TGX1951-3F (**C**) in Zaria (northern Guinea savanna) over a 30‐year (1985–2014) period. SW1: June 1–7; SW2: June 8–14; SW3: June 15–June 21; SW4: June 22–28 June; SW5: June 29–July 5; SW6: July 6–July 12; SW7: July 13–July 19; SW8: July 20–July 26; SW9: July 27–August 2; SW10: August 3–August 9.

**Figure 4:**
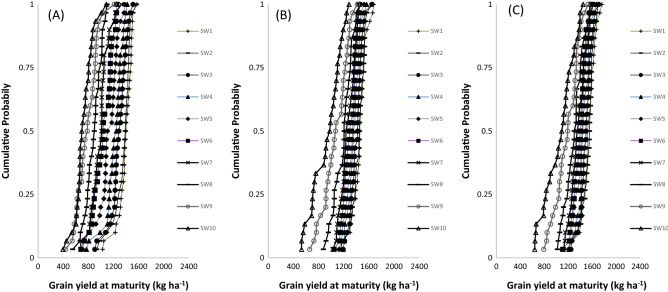
Cumulative function plots for simulated grain yields of TGX1835‐10E (**A**) TGX1904-6F (**B**) and TGX1951-3F (**C**) in Jagiri (southern Guinea savanna) over a 30‐year (1985–2014) period. SW1: June 1–7; SW2: June 8–14; SW3: June 15–June 21; SW4: June 22–28 June; SW5: June 29–July 5; SW6: July 6–July 12; SW7: July 13–July 19; SW8: July 20–July 26; SW9: July 27–August 2; SW10: August 3–August 9.

Cumulative probability distributions (CPD) showing the simulated soybean grain yield of TGX1835‐10E, TGX1904-6F and TGX1951-3F within each site are presented in Figs. 2, 3, 4. In Kano in the SS agro-ecology the CPD shows that each delay in sowing progressively decreases yield of soybean. For variety TGX1835‐10E, sowing between SW1–SW5 resulted in grain yields of above 1200 kg ha^−1^ (range 1210–1639 kg ha^−1^) in 75% of the simulated years while delaying sowing produced yields < 1000 kg ha^−1^ with the lowest yields (< 500 kg ha^−1^) simulated in the late July and August sowings at 75% probability (Fig. 2A). For the medium variety TGX1904-6F, yields above 1200 kg ha^−1^ (range 1229–1440 kg ha^−1^) were simulated in 75% of the years when sowing between SW1–SW3. In this agro-ecology, sowing this variety after the end of June (SW4) produced yields below 1000 kg ha^−1^ (Fig. 2B). The early maturing variety TGX1951-3F showed similar trend with TGX1835‐10E. The sowing windows early June (SW1) to early July (SW5) simulated the highest yields in 75% of the cases, and lowest yields were simulated when sowing was delayed to late July and August (Fig. 2C).

The CPD plot in Zaria in the northern Guinea savanna zone showed that sowing TGX1835‐10E from early June (SW1) to early‐July (SW5) resulted in higher and marginal grain yield ranging from 1434 to 1550 kg ha^−1^ in 75% of the years simulated (Fig. 3A). However, delaying sowing of this variety to SW6 and SW7 reduced grain yield by 12 and 34%, respectively. At this level of probability, delaying sowing of TGX1835‐10E to late July (SW8) and beyond produced yields below 900 kg ha^−1^. Sowing TGX1904-6F from SW1 to SW5 and TGX1951-3F from SW1 to SW6 produced the highest grain yield above 1500 kg ha^−1^ in 75% of the years in Zaria (Fig. 3B,C). Delaying sowing beyond SW5 significantly reduced yield by 10–116% for TGX1904-6F and 18–83% for TGX1951-3F when sowing was delayed beyond SW6.

At Jagiri in the southern Guinea savanna, the CPD plot shows that sowing TGX1835‐10E from early-June to early-July (SW1–SW5) will produce a guaranteed yield of over 1000 kg ha^−1^ in 75% of the years. Sowing this variety beyond early-July (SW5) will reduce yield by over 18–69%. (Fig. 4A). Sowing the variety TGX1904-6F from June 1 (SW1) to late-July (SW8) will produce a yield of 1000–1372 kg at ha^–1^ with high level of probability (P = 0.75) beyond which yield will reduce by 14% for SW9 and 50% for SW10 (Fig. 4B). For TGX1951-3F sowing from early June (SW1) to early August (SW9) will produce yield of 1041–1420 kg ha^–1^ but when sowing is delayed to SW10, the yield reduced by 27% at 75% probability (Fig. 4C).

## Discussion

Soybean is increasingly becoming an important economic crop in the savannas of northern Nigeria. Its yield is however, limited by poor soil fertility and variable rainfall due to climate change. The determination of optimum crop management practices such as optimum sowing windows can provide useful information for strategic planning in the country. However, this process is time consuming and expensive. The use of a dynamic crop simulation model can be an alternative option to estimate yield levels under different growing conditions. Variation in crop yield associated with genotype, environment and management practices can be explained through the use of crop models^27^.

In this study, the statistics for calibration and evaluation of CROPGRO-soybean model indicated good agreements between the model simulated and observed values for soybean phenology and grain yield. This is indicated by low nRMSE and RMSE as well as high d-index values for all measured parameters. Based on these results, it can be concluded that the model was very robust in predicting the critical phenological growth stages and yield of the soybean varieties. This suggests that the CROPGRO-soybean model is a reliable tool for decision making in the production of soybean in the savannas of Nigeria. The accuracy of CROPGRO-soybean model in the study area supports previous research findings in similar environments in Kenya^25^, Nigeria^56^, and Mozambique^57^ in Sub Saharan Africa, which showed good agreement between observed and predicted values for soybean growth and yield using the CROPGRO-Soybean model.

The phenological parameters were accurately predicted with less than 2 days between the simulated and observed values. Wang et al.^58^ suggested that predicting days to flowering is among the important stage for model simulation as time between emergence to flowering determines plant size thereby influencing dry matter production and final crop yield. Robertson et al.^59^ stated that all genotypic variations affecting leaf area development, total dry matter and grain yield will be captured by the model when the phenology is accurately calibrated. The model also performed well in simulating grain yield of all the varieties in the diverse savannas of Nigeria with higher average *d* index above 0.95 for each variety across the two locations. Talacuece et al.^57^ also reported high d-index values of 0.96 and 0.99 for grain yield of the tropical soybean cultivars in Mozambique. The result of the evaluation shows that the accuracy of prediction of all the measured parameters were better with applied P-rates compared with no P application. This agrees with the findings of Naab et al.^60^ who observed high accuracy of prediction with high P-fertilizer rate and lower prediction accuracy under low or no P-fertilizer application when simulating for groundnut growth using CROPGRO-Peanut.

The inter-annual variability for early SWs showed low variability ranging from 6 to 15% compared to delayed SWs which are estimated above 15% of the average grain yield, among the soybean varieties and sites except for Kano site. Our results revealed that early SWs simulated high grain yield while delay SWs led to low yield, which varied among the agro-ecozones. For instance, in Kano, the length of optimal sowing period was estimated at 35 days for the soybean varieties. Sowing from June 1 to July 5 (SW1–SW5) could be more favourable to attain reasonable yields (1366–1763 kg ha^−1^) of the extra-early maturing TGX1835-10E, and early maturing TGX1951-3F (1266–1714 kg ha^−1^). Optimum planting window for TGX1904-6F is late June (SW4) when yields of 1261–1590 kg ha^−1^ can be obtained.

In Zaria (northern Guinea savanna) and Jagiri (southern Guinea savanna) the trend in sowing window was found to be similar with simulated yields showing that, sowing could be extended to July 12. This implies that the length of the optimal sowing window in these two locations is 42 days. Despite the stable and higher rainfall in Jagiri during the growing seasons (Table 2), the simulated average yield in Zaria was higher across the sowing window compared to this location. Generally, the yields in Jagiri were found to be lower which could be attributed to high sand content of the soil resulting to low water holding capacity and nutrient retention. From the results, Zaria was found to be more favourable for soybean production. The soils in Zaria have higher organic matter and clay contents resulting to high water retention (0.543 cm^3^ cm^−3^) and high nutrient content. Tofa et al.^15^ also simulated higher yield of maize in Zaria in the northern Guinea savanna than in Kano in the Sudan savanna and Abuja in the southern Guinea savanna.

The study showed that in Sudan savanna, there is a high probability that higher yields will be obtained when extra-early (TGX1835‐10E) and early (TGX1951-3F) maturing soybean varieties are planted between early June to early-July. Because of short planting window and the uncertainty in rainfall at the beginning of the season coupled with the narrow window of sowing, it is not advisable to plant the medium-maturing variety, TGX1904-6F in Kano in the Sudan savanna. This suggests that farmers in this zone should plant extra-early and early maturing varieties to avoid drastic yield reduction. In northern Guinea savanna, there is a high probability that higher optimum yields will be obtained when all the varieties were sown between early June to early-July except for TGX1951-3F, which also gave optimum yield when sowing was delayed to mid‐July because of its high yield potential. TGX1835-10E and TGX1951-3F were found to have less yield reduction than TGX1904-6F when planted beyond mid-July. This is attributable to the moderate amount of rainfall in the northern Guinea savanna and the long growing season, which allows the earlier maturing varieties to complete their growth cycles when planted late in the season. Although the crop performance in the southern Guinea savanna was lower than in the Sudan savanna and northern Guinea savanna probably due to poor soil fertility, the probability of obtaining optimal yield with later sowing windows was high. Average yields of 1 t ha^−1^ for the extra-early maturing TGX1835-10E and 1.38 t ha^−1^ for the early-maturing TGX1951-3F can be obtained if planting is delayed to July 12 while an average yield of 1.3 t ha^−1^ can be obtained if planting of TGX1904-6F is delayed to July 5. The results also indicated that late sowing beyond mid-July is risky and can leads to a higher yield reduction or crop failure in all the agro-ecologies.

For all the agroecological zones, sowing soybean beyond mid-July significantly reduced grain yield. This may be due to drought stress arising from moisture deficit before the soybean crop complete its life cycle. Rainfalls towards the end of the growing season in the Nigeria savannas are not stable^15^. The rains can cease in September or October before the crops reach maturity which can significantly reduce the yield and yield components of soybean. Drought stress has been reported to significantly reduce the yield of soybean. Therefore, information on suitable planting window that will help farmers plant their crops and avoid end of season drought will be useful.

Using the CERES-Maize model, Tofa et al.^15^ reported reduced grain yield of maize as a result of delay in sowing dates beyond late July for medium-maturing maize in the Nigeria savannas. Beah et al.^61^ used the APSIM model to simulate optimum sowing window of early and medium-maturing maize varieties in the Nigeria savannas. Their results showed that for optimum grain yield for both early and medium maize varieties, sowing should be done between June 15 to 28 in all the agro-ecologies.

Sowing date is an important factor influencing soybean growth and yield. The growth and yield responses of soybean to sowing date depend on the cultivar, environment and management practice^62^. There was variation in the performance of soybean among the agro‐ecologies in the study area. This could be attributed to variation in the maturity periods, soil and weather conditions (temperature, rainfall and solar radiation) in the study area. Lin et al.^63^ stated that soil, climate and crop variety are the most important factors that affect crop production. For all the varieties, averaged mean grain yield across sowing windows were generally higher in the northern Guinea savanna than the other agro‐ecologies irrespective of the length of growing season. This is probably due to the good soil conditions in this agro‐ecology where the clay and organic carbon contents are higher (Table 1) than in the other agro-ecologies where the soils were sandy. Due to the variability in onset of rainfall in the Nigeria savannas, sowing window recommendations should be made with caution. Most often establishment of rains vary significantly between first week of June to early July due to climate change. This variability influences commencement of sowing of crops in the region.

## Conclusion

The DSSAT CROPGRO-Soybean model was calibrated and evaluated for the simulation of sowing windows of three tropical soybean varieties in the Nigeria savannas. The model calibration and evaluation results showed that there was good agreement between the simulated and observed values for phenology, growth, and yield parameters of the soybean varieties This suggests that the CSM-CROPGRO-Soybean model was able to accurately simulate the growth and yield of soybean in the Nigeria savannas. This study also showed the potential of the model to serve as a tool for determining optimum sowing window for soybean in the savannas. The simulation results show significant differences among the agro-ecologies and the soybean varieties in terms of grain yield. The results revealed higher yields are simulated among the soybean varieties in northern Guinea than in southern Guinea and Sudan savanna agro-ecological zones probably due to the high clay content in the northern Guinea savanna leading to high water and nutrient retention capacity. The least grain yields were simulated for the southern Guinea savanna than for the other zones despite higher rainfall and longer growing season probably due to the poor soil fertility in this zone. In addition, higher yield was simulated for the variety TGX1951-3F than for TGX1904-6F and TGX1835-10E in all the agro-ecozones. Our results revealed that length of sowing windows is dependent on agro-ecology and soybean variety. Sowing from June 1 to July 5 (SW1–SW5) produced optimal yield of TGX1951-3F and TGX1835-10E in Kano in the SS zone. Delaying sowing beyond July 5 will results in significant yield loss for all the varieties. The cultivation of TGX1904-6F is not recommended for the Sudan savanna zone because of its late growing cycle. In Zaria (northern Guinea savanna) and Jagiri (southern Guinea savanna) the simulated results show that, sowing could be extended to July 12 for all the varieties. Generally sowing beyond July 12 for all the varieties significantly reduced grain yield in all the agro-ecozones which may be due to drought stress at the end of growing season which coincided with grain-filling period.

## Data Availability

The data that support the findings of this study are available from the corresponding author upon reasonable request.
